# Mitochondrial Pathway Mediates the Antileukemic Effects of *Hemidesmus Indicus*, a Promising Botanical Drug

**DOI:** 10.1371/journal.pone.0021544

**Published:** 2011-06-28

**Authors:** Carmela Fimognari, Monia Lenzi, Lorenzo Ferruzzi, Eleonora Turrini, Paolo Scartezzini, Ferruccio Poli, Roberto Gotti, Alessandra Guerrini, Giovanni Carulli, Virginia Ottaviano, Giorgio Cantelli-Forti, Patrizia Hrelia

**Affiliations:** 1 Department of Pharmacology, Alma Mater Studiorum, University of Bologna, Bologna, Italy; 2 Department of Evolutionary and Experimental Biology, Alma Mater Studiorum, University of Bologna, Bologna, Italy; 3 Department of Pharmaceutical Sciences, Alma Mater Studiorum, University of Bologna, Bologna, Italy; 4 Department of Biology and Evolution, Agro-Technological and Pharmaceutical Resources (Agri-Unife), University of Ferrara, Ferrara, Italy; 5 Division of Hematology, Department of Oncology, Transplants and New Technologies in Medicine, University of Pisa, Pisa, Italy; The University of Kansas Medical Center, United States of America

## Abstract

**Background:**

Although cancers are characterized by the deregulation of multiple signalling pathways, most current anticancer therapies involve the modulation of a single target. Because of the enormous biological diversity of cancer, strategic combination of agents targeted against the most critical of those alterations is needed. Due to their complex nature, plant products interact with numerous targets and influence several biochemical and molecular cascades. The interest in further development of botanical drugs has been increasing steadily and the FDA recently approved the first new botanical prescription drug. The present study is designed to explore the potential antileukemic properties of *Hemidesmus indicus* with a view to contributing to further development of botanical drugs. *Hemidesmus* was submitted to an extensive *in vitro* preclinical evaluation.

**Methodology/Principal Findings:**

A variety of cellular assays and flow cytometry, as well as a phytochemical screening, were performed on different leukemic cell lines. We have demonstrated that *Hemidesmus* modulated many components of intracellular signaling pathways involved in cell viability and proliferation and altered the protein expression, eventually leading to tumor cell death, mediated by a loss of mitochondrial transmembrane potential and increased Bax/Bcl-2 ratio. ADP, adenine nucleotide translocator and mitochondrial permeability transition pore inhibitors did not reverse *Hemidesmus*-induced mitochondrial depolarization. *Hemidesmus* induced a significant [Ca^2+^]_i_ raise through the mobilization of intracellular Ca^2+^ stores. Moreover, *Hemidesmus* significantly enhanced the antitumor activity of three commonly used chemotherapeutic drugs (methotrexate, 6-thioguanine, cytarabine). A clinically relevant observation is that its cytotoxic activity was also recorded in primary cells from acute myeloid leukemic patients.

**Conclusions/Significance:**

These results indicate the molecular basis of the antileukemic effects of *Hemidesmus* and identify the mitochondrial pathways and [Ca^2+^]_i_ as crucial actors in its anticancer activity. On these bases, we conclude that *Hemidesmus* can represent a valuable tool in the anticancer pharmacology, and should be considered for further investigations.

## Introduction

Cancer is a complex disease characterized by multiple genetic and molecular alterations involving transformation, deregulation of apoptosis, proliferation, invasion, angiogenesis and metastasis [1]. It now appears that, for many cancers, multiple, redundant aberrant signaling pathways are at play as a result of genetic perturbations at different levels. Recent studies observe that in any given type of cancer 300–500 normal genes have been modified to result in the cancerous phenotype [2]. Although cancers are characterized by the deregulation of multiple signalling pathways at multiple steps, most current anticancer therapies involve the modulation of a single target. Because of the enormous biological diversity of cancer, strategic combination of agents targeted against the most critical of those alterations is needed. In addition, due to mutation in the target, treatment of cancer cells with a mono-targeted agent may induce adaptive resistance to a mono-targeted agent, but resistance is less likely if there are multiple targets [2], [3]. Various cell signalling network models indicate that partial inhibition of a number of targets is more effective than the complete inhibition of a single target [2]. Multi-targeted drugs hit multiple targets. An example of multi-targeted drug is sunitinib as it targets c-KIT, but it also has activity against receptors for vascular endothelial growth factor, platelet-derived growth factor and the FMS-like tyrosine kinase 3 (FTLT3). In addition to multi-targeted therapeutics, multicomponent therapeutics is also proposed [4].

Due to their complex nature, accumulating evidence suggests that plant products interact with numerous recent targets, which strengthens the view that they influence numerous biochemical and molecular cascades [5]. They are also relatively safe and affordable in most cases.

In recent years, the interest in further development of botanical drug products has been increasing steadily. Recently, the FDA approved the first botanical drug, a water extract of green tea leaves for perianal and genital condyloma. Unlike most small-molecule drugs that are comprised of a single chemical compound, the FDA-approved drug contains a mixture of known and possibly active compounds [6]. It is the first new botanical prescription drug approved by the FDA since the publication of the FDA’s industry guidelines for botanical drug products in June 2004. Of note, as specified in the FDA’s guidelines, the term *botanicals* does not include highly purified substances derived from botanical sources [7]. However, the approval of the first botanical drug shows that new therapies from natural complex mixtures can be developed to meet current FDA standards of quality control and clinical testing.

In the last few years, interest in developing botanical drugs escalated. The number of submissions increased rapidly from 5–10 per year in 1990–1998 to an average of 22 per year in 1999–2002 and nearly 40 per year in 2003–2007 [6]. In the United States, there are about 10 to 20 botanical drugs that are going through serious clinical development [8]. Among the therapeutic areas, the number of botanical products submitted to the FDA was particularly high for cancer and related conditions. These data indicate a growing interest in several therapeutic areas towards a rigorous clinical evaluation of botanical drugs, with a focus on indications where there is a clear medical need for new treatments (*e.g.*, cancer) [6].

*Hemidesmus indicus* Linn. R. Br. (Family Asclepiadaceae) has been found to exhibit many biological activities, such as antitumor, anti-inflammatory, antioxidant, antimicrobial, hepatoprotective, nephroprotective, otoprotective [9]–[13]. Despite its different biological effects, the extensive phytochemical investigations and its past admittance in the British Pharmacopoeia [14]–[18], *Hemidesmus* lacks systematic scientific evaluation and its anticancer mechanisms remain elusive.

The present study was designed to explore the potential antileukemic properties of *Hemidesmus indicus* with a view to contributing to further development of herbal or botanical drug products derived from traditional preparations. *Hemidesmus* was submitted to an extensive *in vitro* preclinical evaluation. In particular, we tested the decoction of the plant roots, which represents the most used form in the traditional medicine. First of all, we performed a phytochemical screening of the decoction. We then investigated its antileukemic effects, also in association with some conventional anticancer drugs, and assessed the molecular mechanisms involved in its cytostatic and cytotoxic effects through an extensive flow cytometric investigation. Since a key event in the antileukemic activity of *Hemidesmus* was [Ca^2+^]_i_ rises, we explored the underlying mechanisms through the use of different inhibitors. Finally, we tested *Hemidesmus* on cells isolated from acute myeloid leukemia (AML) patients.

## Methods

### Ethics statement

The described study was approved by the Comitato Etico e Sperimentazione del Farmaco dell'Azienda Ospedaliero-Universitaria Pisana and written informed consent was obtained from the patients. All clinical investigation was conducted according to the principles expressed in the Declaration of Helsinki.

### Plant decoction preparation

*Hemidesmus indicus* (voucher #MAPL/20/178) was collected from Ram Bagh (Rajasthan, India), and authenticated by Dr. MR Uniyal, Maharishi Ayurveda Product Ltd., Noida, India. The plant decoction was prepared according to the method described in the Ayurvedic Pharmacopoeia of India [19]. Briefly, 10 g of grinded roots of *Hemidesmus* were mixed with 300 mL of boiling water, allowing the volume of water to reach 75 mL. The decoction was filtered, lyophilized, aliquoted and stored at room temperature. Immediately before the assays, the samples were resuspended in water and centrifuged at 2000 rpm to discard any insoluble material. Eventually, samples were sterile-filtered when necessary.

### HPLC analysis of plant decoction

HPLC analysis was performed to quantify the main phytomarkers of *Hemidesmus* samples, namely 2-hydroxy-4-methoxybenzaldehyde, 3-hydroxy-4-methoxybenzaldehyde and 2-hydroxy-4-methoxybenzoic acid. The reference compounds (all obtained from Sigma, St. Louis, MO, USA) were used as external standards to set up and calculate appropriate calibration curves. The experimental conditions were performed using a Jasco modular HPLC (Tokyo, Japan, model PU 2089) coupled to a diode array apparatus (MD 2010 Plus) linked to an injection valve with a 20 µL sampler loop. The column used was a Tracer Extrasil ODS2 (25×0.46 cm, i.d., 5 mm) with a flow rate of 1.0 mL/min. The mobile phase consisted of solvent solution B (methanol) and A (water/formic acid  = 95∶5). The gradient system adopted was characterized by five steps: 1, isocratic, B/30 for 15 min; 2, B raised progressively from 30% to 40% at 20 min; 3, B then raised to 60% at 50 min; 4, B achieved 80% at 55 min and 5, 100% at 60 min. Injection volume was 40.0 µL. Chromatograms were recorded, and peaks from *Hemidesmus* samples were identified by comparing their UV spectra and retention time with those from the pure standards. The identity was also confirmed by ^1^H NMR on the enriched fraction of the compounds obtained by soxhlet extraction in CHCl_3_/EtOH 1∶1.

Peak area was calculated by integration using dedicated Borwin software (Borwin ver. 1.22, JMBS Developments, Grenoble, France).

### Standard solution and calibration procedure

Individual stock solutions of 2-hydroxy-4-methoxybenzaldehyde, 3-hydroxy-4-methoxybenzaldehyde and 2-hydroxy-4-methoxybenzoic acid were prepared in water. Six different calibration levels were prepared within the following range: 2.0–20.0 µg/mL for 2-hydroxy-4-methoxybenzaldehyde, 1.5–40.0 µg/mL for 3-hydroxy-4-methoxybenzaldehyde, and 1.0–100.0 µg/mL for 2-hydroxy-4-methoxybenzoic acid. Each calibration solution was injected into HPLC in triplicate. Regression analysis of peak area of the analytes *versus* the related concentrations provided the calibration graphs.

The analysis of the decoction (31 mg/mL) was carried out under the same experimental conditions and the concentration of the three components was determined by the obtained calibration graphs. At least three different batches of *Hemidesmus* were tested.

### Leukemic cell lines

Human leukemia Jurkat (acute T lymphoblastic leukemia), CEM (acute T lymphoblastic leukemia), HL-60 (acute promyelocytic leukemia), REH (non-T, non-B lymphoblastic leukemia) and KU812F (chronic myeloblastic leukemia) cell lines were grown in suspension and propagated in RPMI 1640 supplemented with 10% (Jurkat, CEM, REH, KU812F) or 20% (HL-60) heat-inactivated bovine serum, 1% antibiotics (all obtained from Sigma). To maintain exponential growth, the cultures were divided every third day by dilution to a concentration of 1×10^5^ cells/mL. Cells were treated with different concentrations of *Hemidesmus* (0.0–3.1 mg/mL) (prepared from a stock solution of 31 mg/mL) for different times at 37°C.

### Patients

The characteristics of the patients studied are given in Table 1. The diagnosis of leukemia was established by combination of morphological, immunological, cytogenetic and molecular methods, which were applied to peripheral blood samples. The immunological assays were made by fluorochrome-conjugated monoclonal antibodies and analysis by a three-laser (488, 633, 405 nm)-equipped flow cytometer (FacsCanto II, Becton Dickinson, San Jose, CA, USA). A six-color method was applied; therefore the following fluorochrome combination was used: fluorescein isothiocyanate, phycoerythrin, peridinin chlorophyll protein complex, phycoerythrin-cyanine 7, allophycocyanin, allophycocyanin-cyanine 7. A wide panel of monoclonal antibodies was used that always included: CD45, CD13, CD33, CD34, CD117, HLA-DR, CD4, CD14, CD64, CD38, MPO, CD11b, CD16, CD15, CD56, CD7, CD19 (all from Becton Dickinson). Cytogenetic analysis was made by standard banding methods and by fluorescence *in situ* hybridization methods. The molecular methods, carried out by PCR, included the following fusion genes: AML1/ETO, CBFβ/MYH11, and BCR-ABL. In addition, FLT3/internal tandem duplication (ITD) was also investigated. According to current prognostic criteria, four risk groups (low, intermediate, intermediate-high, high) were defined. Patients were studied at the time of diagnosis; one patient was studied during him first relapse.

**Table 1:** Clinical features of patients.

Diagnosis	Sex	Age	FAB classification	WBC^a^	Immunophenotype	Karyotype	Molecular biology	Risk
AML-1	M	78	M5	15 000	CD33+, CD13+, CD38+, CD15+	46,XY 20%; 47,XY;+8 80%	Negative	Intermediate-high
AML-2	F	66	M4	37 000	HLA-DR+, CD13+/−, CD33+, CD64+, CD14+/−, CD4+, CD11b+, CD15+, CD38+	46,XX	FLT3 positive	Intermediate
AML-3	M	85	M5	38 000	CD13+, CD33+, CD14+, CD64+, CD56+, CD15+, CD11b+	46,XY	Negative	Intermediate
AML-4	M	62	M4	30 000	CD34+, CD33+, CD13+, CD117+/−, CD7+, CD38+, CD15 +/−	46,XY	FLT3 positive/ITD^b^	High

a WBC: number of cells/mm^3^ whole blood;

b internal tandem duplication.

### Preparation of leukemic cells

Peripheral blood samples of patients were collected in tubes containing preservative-free heparin. Leukemic cells were obtained by Ficoll-Histopaque density gradient centrifugation. Interphase mononuclear cells were recovered, washed twice with phosphate-buffered saline and then resuspended in RPMI 1640 medium (Sigma) containing 15% heat-inactivated bovine serum. The samples always contained >95% blasts.

### Flow cytometry

All flow cytometric procedures were performed with a Guava EasyCyte Mini flow cytometer (Guava Technologies-Millipore, Hayward, CA, USA). Approximately 5,000 events (cells) were evaluated for each sample. In all cytofluorimetric determinations, cell debris and clumps were excluded from the analysis by gating.

### Cell viability

Cells were treated with different concentrations of *Hemidesmus* for one cell cycle. Viability was determined immediately after the end of treatment by flow cytometry. Briefly, cells were mixed with an adequate volume of Guava ViaCount Reagent (containing propidium iodide, Guava Technologies) and allowed to stain 5 min at room temperature. IC_50_ (inhibitory concentration causing cell toxicity by 50% following one cell-cycle exposure) was calculated by interpolation from dose-response curve.

### Detection of apoptosis

After 24 h of treatment with different concentrations of *Hemidesmus* alone or in combination with methotrexate (0.05–0.25 µM, Sigma), 6-thioguanine (3.75–30 µM, Sigma) or cytarabine (0.10–1.25 µM, Sigma), aliquots of 2.0×10^4^ cells were stained with 100 µL of Guava Nexin Reagent (containing ANNEXIN-V-phycoerythrin and 7-amino-actinomycin D) and incubated for 20 min at room temperature in the dark. Samples were then analyzed by flow cytometry. Camptothecin 2 µM was used as positive control.

The analysis was also performed on Jurkat cells treated with 2-hydroxy-4-methoxybenzaldehyde (2.5 µg/mL), 3-hydroxy-4-methoxybenzaldehyde (18.5 µg/mL) or 2-hydroxy-4-methoxybenzoic acid (22.0 µg/mL).

### Detection of caspase-8 and caspase-3 activity by flow cytometry

Active caspases’ detection employed an affinity label methodology, using the caspase-8-preferred substrate leucine-glutamic acid-threonine-aspartic acid (LETD) or the caspase-3-preferred substrate amino acid sequence aspartic acid-glutamic acid-valine-aspartic acid (DEVD) linked to a fluoromethylketone (FMK) moiety, which reacts covalently with the catalytic cysteine residue in the active enzymatic center. A 6-carboxyfluorescein (FAM) group linked to LETD- or DEVD-FMK was used as a reporter. After 24 h of treatment with *Hemidesmus* 0.93 mg/mL, cells were stained with 10 µL of freshly prepared 10X working dilution FAM-LETD-FMK (Guava Technologies) or 10 µL of freshly prepared 30X working dilution FAM-DEVD-FMK solution (CHEMICON International, Temecula, CA, USA) and incubated for 1 h at 37°C, protecting tubes from light. After washing, cells were resuspended in 150 µL of 7-aminoactinomycin D diluted 1∶200 in 1X working dilution wash buffer (Guava Technologies), incubated for 5 min at room temperature in the dark, and analyzed via flow cytometry. Camptothecin 2 µM was used as positive control.

### Measurement of mitochondrial potential

Mitochondrial potential was assessed by using JC-1 (5,5′,6,6′-tetrachloro-1,1′,3,3′-tetraethylbenzimidazol-carbocyanine iodide). After 24 h of treatment with *Hemidesmus* 0.93 mg/mL, 200 µL of cell suspension were treated with 4 µL of 50X staining solution (Guava Technologies), containing JC-1 and 7-amino-actinomycin D. Cells were incubated for 30 min at 37°C and analyzed via flow cytometry. Valinomycin 0.09 µM was used as positive control. The experiments were also performed in the presence of bongkrekic acid (BA, 20 µM, Sigma) for 2 h, carboxyatractyloside (CATR, 20 µmol/L, Sigma) for 90 min, ADP (500 µM) plus oligomycin (20 µg) for 1 h, or cyclosporine A (Cyc A, 1 µM, Sigma) for 1 h. The cultures were preincubated with the above reported compounds for the indicated times then cultured with and without *Hemidesmus*.

### Analysis of cytochrome c release

Mitochondrial cytochrome c release was monitored during cell death of digitonin-permeabilized cells immunolabeled for cytochrome c. After triggering of apoptosis by *Hemidesmus* treatment, we determined the fraction of cells that have not yet released their mitochondrial cytochrome c and were still highly fluorescent, as well as the fraction of apoptotic cells that have already released their mitochondrial cytochrome c and, therefore, were much less fluorescent.

Cells (1×10^6^) were harvested and treated with 1 mL digitonin (100 µg/mL, Sigma) for 5 min on ice. Cells were fixed in formaldehyde 4% for 20 min at room temperature, washed three times in PBS 1x and incubated in incubation buffer (0.5 g BSA in 100 mL PBS 1x) for 1 h. The cells were incubated overnight at 4°C with 1∶200 anti-cytochrome c monoclonal antibody (clone 7H8.2C12, BD Pharmingen, San Diego, CA, USA) in incubation buffer, washed three times and incubated for 1 h at room temperature with fluorescein isothiocyanate-labeled secondary antibody (1∶100, Sigma). The cells were then analyzed to quantify fluorescein isothiocyanate binding by flow cytometry. Mean fluorescence intensity values were calculated. Non-specific binding was excluded by gating around those cells which were labeled by the fluorescein isothiocyanate-conjugate isotype control.

### Measurement of intracellular Ca^2+^ ([Ca^2+^]_i_)

[Ca^2+^]_i_ was measured using the cell-permeable Ca^2+^-sensitive fluorescent dye Fluo-3 acetoxymethyl ester. This dye freely permeates the surface membrane but, following hydrolysis by intracellular esterases, is trapped in cells as Fluo-3. The fluorescence intensity of Fluo-3 is enhanced after it binds to [Ca^2+^]_i_ and depends on the free calcium concentration [20]. Cells were incubated for 20 min at room temperature with 4 µM Fluo-3 acetoxymethyl ester diluted in Krebs-Ringer buffer [10 mM D-glucose, 120 mM NaCl, 4.5 mM KCl, 0.7 mM Na_2_HPO4, 1.5 mM NaH_2_PO_4_, and 0.5 mM MgCl_2_ (pH 7.4 at 37°C); Sigma]. After washing, the cultures were treated with the indicated concentration of *Hemidesmus*. At the end of incubation, cells were washed in 5 ml of Ca^2+^-free PBS at 37°C, resuspended in 1 ml of Ca^2+^-free PBS at 37°C and analyzed immediately by flow cytometry.

The experiments were also performed in the presence of nifedipine (10 µmol/L, Sigma), econazole (3 µM, Sigma), thapsigargin (1 µM, Sigma), aristolochic acid (50 µM, Sigma). The cultures were preincubated with the above reported compounds for 10 min then cultured with and without *Hemidesmus* for 10 min. The experiments were performed in Ca^2+^-containing medium (RPMI 1640).

### Cell proliferation

Carboxyfluorescein diacetate succinimidyl ester diffuses freely into cells where intracellular esterases cleave off the acetate groups, converting it to a fluorescent, membrane-impermeant dye. The dye is equally distributed between daughter cells due to covalent crosslinking to proteins through its succinimidyl groups. The stain is long lived, allowing the resolution of at least three or four cycles of cell division. Propidium iodide is then added to distinguish the live from the dead cells. Through the use of differential staining by the two fluorescent dyes, live and dead proliferated and unproliferated cells can be distinguished. 25×10^6^ cells were incubated with carboxyfluorescein diacetate succinimidyl ester for 15 min at 37°C. After three washes, cell concentration was adjusted to 1×10^6^/mL with complete medium. Cells were treated with different concentrations of *Hemidesmus* for 24 h. After incubation, cells were treated with 5 µL of cell growth propidium iodide reagent (Guava Technologies), incubated in the dark at room temperature for 5 min, and analyzed via flow cytometry.

### Cell-cycle distribution

Cells were treated with different concentrations of *Hemidesmus* for 8, 24 and 48 h, and then fixed with ice-cold 70% ethanol. After washing, cultures were resuspended in 200 µL Guava Cell Cycle Reagent (containing propidium iodide), incubated at room temperature for 30 min, shielded from light, and analyzed via flow cytometry.

### Analysis of cell-cycle and apoptotic proteins

After treatment with *Hemidesmus indicus* 0.93 mg/mL for 24 h, 1×10^6^ cells were fixed and permeabilized by 2% of paraformaldehyde in PBS 1X and 90% of cold methanol. They were then incubated with fluorescein isothiocyanate cyclin A (10∶100, Beckman Coulter, Brea, CA, USA), p21 (2∶100, Abcam, San Francisco, CA, USA), cyclin E (1∶100, Abcam), CDK2 (0.6∶100, Abcam), Bax (1∶100, Santa Cruz Biotechnology, Santa Cruz, CA, USA), Bcl-2 (1∶100, Santa Cruz Biotechnology), fluorescein isothiocyanate 85 kDa fragment of cleaved poly ADP-ribose polymerase (1∶100, PARP, Invitrogen), or isotype-matched negative control (1∶100, e-Bioscience, San Diego, CA, USA) antibodies. The cells (except those stained with cyclin A and PARP) were washed and incubated with fluorescein isothiocyanate-labeled secondary antibody (1∶100, Sigma). The cells were then analyzed to quantify fluorescein isothiocyanate binding by flow cytometry. Mean fluorescence intensity values were calculated. Non-specific binding was excluded by gating around those cells which were labeled by the fluorescein isothiocyanate-conjugate isotype control.

### Statistical analysis

All results are expressed as the mean ± SEM. Differences among treatments were evaluated by one-way or two-way ANOVA, followed by Dunnett or Bonferroni *post*-test, using GraphPad InStat version 4.00 for Windows 95 (Graphpad Prism, San Diego, CA, USA). *P*<0.05 was considered significant.

Interactions between *Hemidesmus* and anticancer drugs were classified using the combination index (CI). CI analysis provides qualitative information on the nature of drug interaction, and CI, a numerical value calculated as described in the following equation, also provides a quantitative measure of the extent of drug interaction [21].
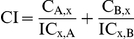



C_A,x_ and C_B,x_ are the concentrations of drug A and drug B used in combination to achieve ×% drug effect. IC_x,A_ and IC_x,B_ are the concentrations for single agents to achieve the same effect. A CI of less than, equal to, and more than 1 indicates synergy, additivity, and antagonism, respectively.

## Results

### Decoction of Hemidesmus indicus contains 2-hydroxy-4-methoxybenzaldehyde, 3-hydroxy-4-methoxybenzaldehyde and 2-hydroxy-4-methoxybenzoic acid

The real sample represented by a decoction of *Hemidesmus indicus* analysed by the applied HPLC method was found to contain 2-hydroxy-4-methoxybenzaldehyde, 3-hydroxy-4-methoxybenzaldehyde and 2-hydroxy-4-methoxybenzoic acid. Linearity, limit of detection (LOD), limit of quantification (LOQ) and recovery were excellent. In particular, the correlation coefficients (r^2^) were found to be >0.999 for the three considered analytes. The sensitivity was estimated by dilution of standard solutions. The LOQ was reasonably assumed as the lowest calibration point for each of the considered phytomarkers. The LODs were found to be 0.53 µg/mL, 0.80 µg/mL and 0.30 µg/mL for 3-hydroxy-4-methoxybenzaldehyde, 2-hydroxy-4-methoxybenzaldehyde and 2-hydroxy-4-methoxybenzoic acid, respectively.

The recovery was estimated by spiking at 10 µg/mL level of concentration of each of the compounds and resulted to be within 96 – 101%.

The amounts found in the decoction (31 mg/mL) were: 2.5±0.1 µg/mL for 2-hydroxy-4-methoxybenzaldehyde, 18.5±0.3 µg/mL for 3-hydroxy-4-methoxybenzaldehyde, 22.2±0.5 µg/mL for 2-hydroxy-4-methoxybenzoic acid. The analyses were performed on the decoction obtained from three different batches and the difference among the batches in the phytomarker content resulted not significant (data not shown).

### Hemidesmus indicus decreases cell viability

**Figure 1:**
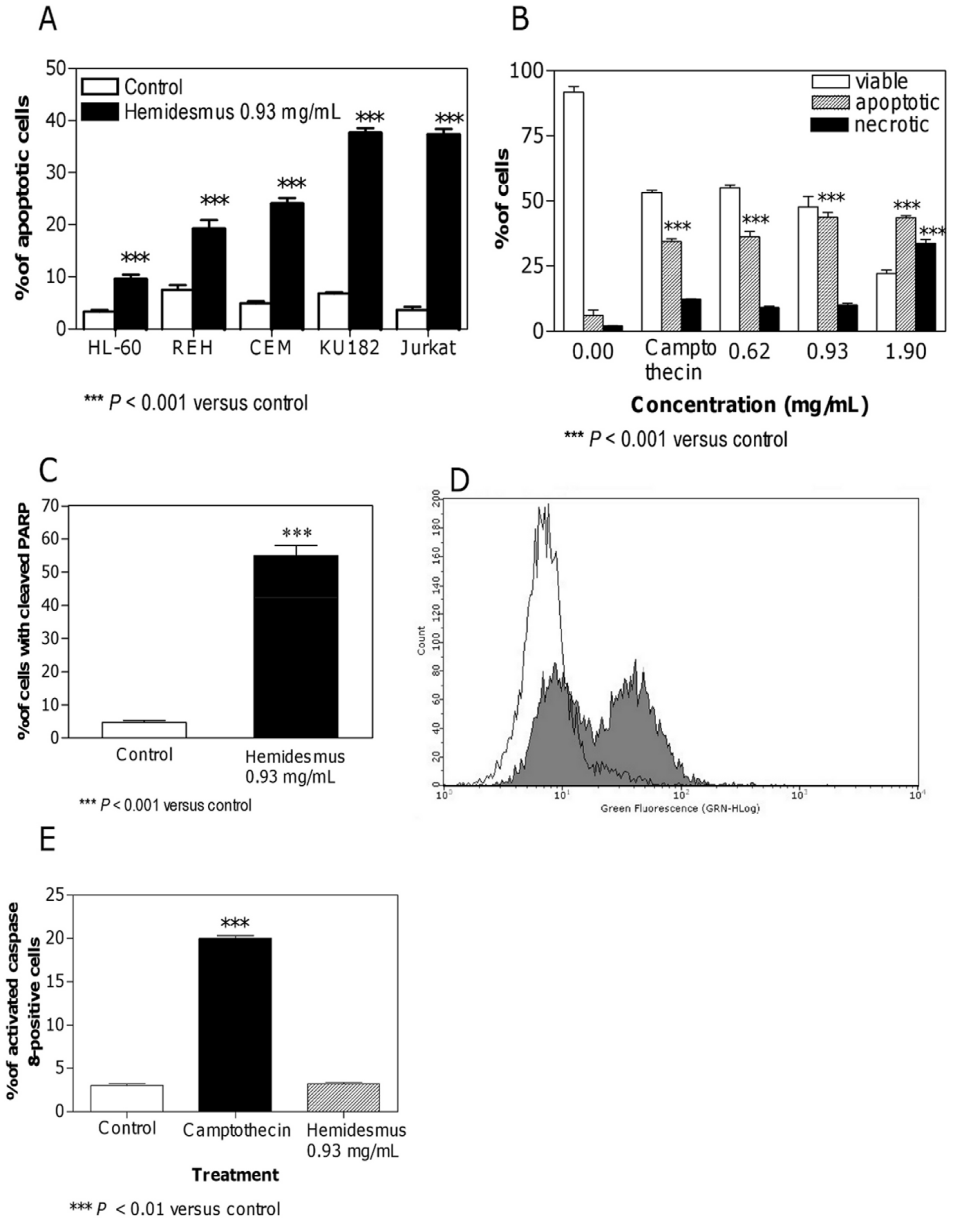
Induction of apoptosis by *Hemidesmus indicus.* Fraction of viable, apoptotic and necrotic cells in different leukemic cell lines (A), in Jurkat cells (B), cleavage of PARP (C–D) and activation of caspase-8 (E) following treatment with *Hemidesmus* for 24 h. Data are means ± SEM of four independent experiments.

*Hemidesmus* reduced cell viability in all tested cell lines (data not shown). Based on the cytotoxicity results, cells were treated with concentrations of *Hemidesmus indicus* similar or smaller than the IC_50_ (0.62, 0.93 and 1.90 mg/mL) for 24 h. *Hemidesmus* treatment induced apoptosis at all the concentrations tested and in all tested cell lines (Figure 1A). For example, after treatment of Jurkat cells with 0.62 mg/mL of *Hemidesmus*, the incidence of apoptotic cells was 36% (*versus* 6% in the control). They increased to 44% after treatment with the highest doses studied (Figure 1B). However, 1.90 mg/mL of *Hemidesmus* induced a significant increase of necrotic cells (Figure 1B). Similar results were recorded for the other cell lines (Figure 1A). Since the highest increase in apoptotic cells with respect to the control was seen in Jurkat cells, the following analyses were conducted in this cell line. For excluding necrotic events, concentrations of up to 0.93 mg/mL were used.

No induction of apoptosis was observed in Jurkat cells following treatment with 2-hydroxy-4-methoxybenzaldehyde, 3-hydroxy-4-methoxybenzaldehyde or 2-hydroxy-4-methoxybenzoic acid (data not shown).

### *Hemidesmus* indicus affects caspase-3 but not caspase-8 activity and activates PARP

Caspase-3 activity was significantly increased in cells treated with *Hemidesmus* (0.93 mg/mL). The percentage of activated caspase-3 cells in non treated cultures was about 3% which was increased to 9% in cells treated with *Hemidesmus* (*P<*0.01) (data not shown). An important reporter for caspase-3 activation is PARP. *Hemidesmus* (0.93 mg/mL) induced PARP cleavage in about 55% of treated cells (Figure 1C). Figure 1D shows a representative cytogram where two well-defined cell populations are distinguishable in *Hemidesmus*-treated cells after labeling with fluorescein isothiocyanate 85 kDa fragment of cleaved PARP. Only one population characterized by a lower fluorescence intensity (white histogram) was recorded in untreated cells.

Unstimulated Jurkat T cells incubated with FAM-LETD-FMK generated a low detectable fluorescence signal, indicating that levels of active caspase-8 were low in these cells. Caspase-8 activity increased after treatment with the positive control camptothecin but remained at basal levels after treatment with 0.93 mg/mL of *Hemidesmus* (Figure 1E).

### *Hemidesmus* indicus disrupts mitochondrial transmembrane potential

**Figure 2:**
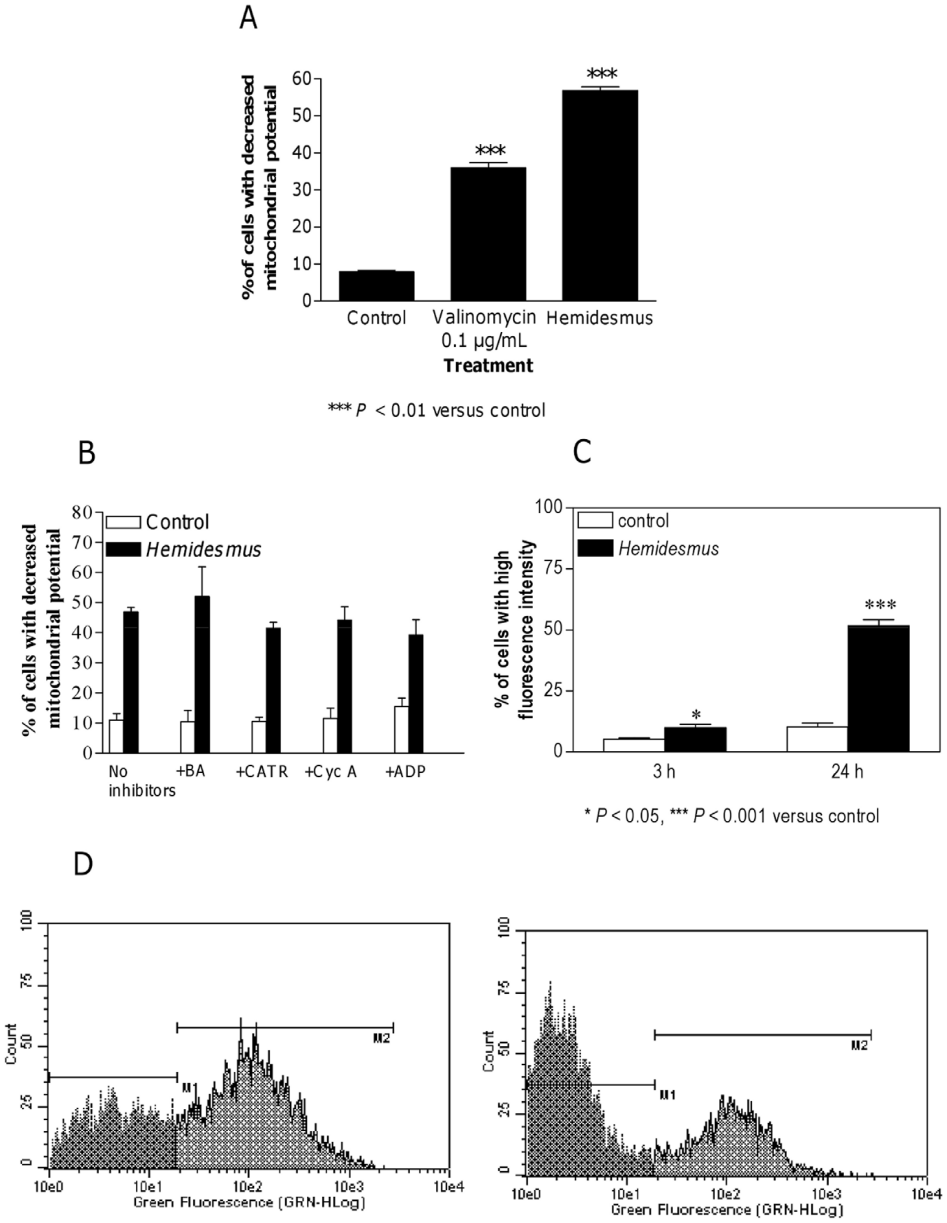
Fraction of cells with decreased mitochondrial potential and increased [Ca^2+^]_i_ after *Hemidesmus* treatment. Alterations in mitochondrial membrane permeability in absence (A) and presence (B) of inhibitors following treatment with *Hemidesmus* 0.93 mg/mL. Fraction of cells with increased [Ca^2+^]_i_ following 3 or 24 h culture in the absence or presence of *Hemidesmus* (0.93 mg/mL) (C), flow cytometric analysis of [Ca^2+^]_i_ following 24 h culture in the absence or presence of *Hemidesmus* (0.93 mg/mL) (D). M1 and M2 indicate the two different populations characterized by a different mean fluorescence intensity values. Data are means ± SEM of four independent experiments.

Treatment of Jurkat cells with 0.93 mg/mL of *Hemidesmus indicus* resulted in a significant break-down of the mitochondrial membrane potential. The effect of *Hemidesmus* resulted even greater than that observed for valinomycin, used as positive control (Figure 2A).

**Figure 3:**
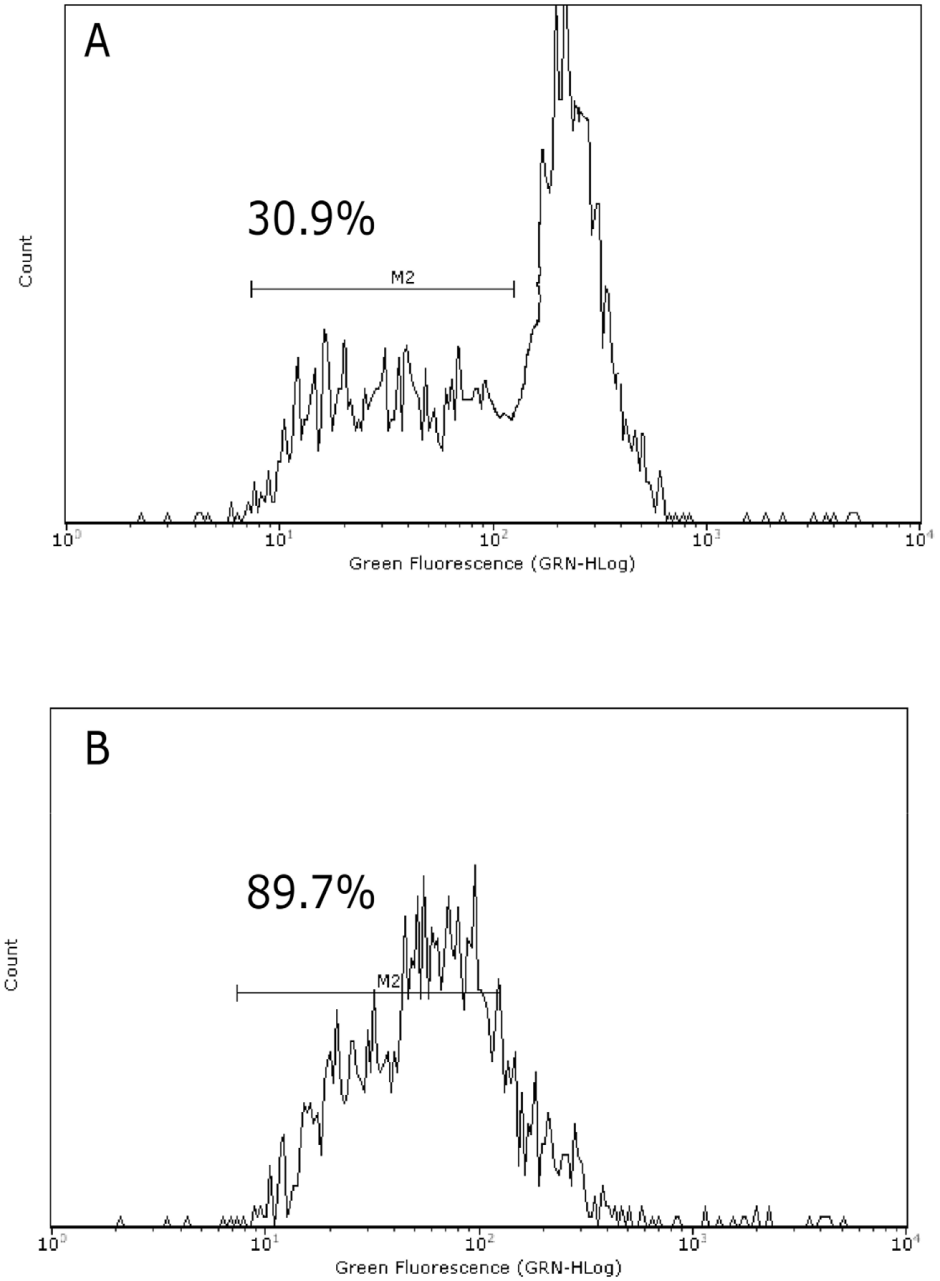
Fluorescence histograms of immunolabeled cytochrome c in cells untreated (A) or treated (B) with *Hemidesmus* (0.93 mg/mL). Note the decrease of the highly fluorescent cell population in region 1 (M1) and the progressive increase of the nonfluorescent cell population in region 2 (M2). M1 and M2, regions arbitrarily defined to limit the population of cells with high and low fluorescence, respectively. Numbers at top of M1 and M2 regions are percentages of cells in each region. Histograms are representatives of three independent experiments.

Cytochrome c release from mitochondria to cytosol is a hallmark of apoptosis and is used to characterize the mitochondria-dependent pathway of this type of cell death. After treatment with *Hemidesmus* (0.93 mg/mL), the fraction of cells that retained their mitochondrial cytochrome c, or the highly fluorescent cells, gradually decreased and emerged as a population of low fluorescent cells (Figure 3).

Since the mitochondrial pathway resulted clearly involved in the proapoptotic activity of *Hemidesmus*, we investigated whether classic adenine nucleotide translocator modulators, such as CATR and BA, or Cyc, which targets cyclophilin D in the matrix, could interfere with the action of *Hemidesmus* on mitochondrial depolarization. Mitochondrial depolarization induced by *Hemidesmus* was not specifically inhibited by BA, CATR or Cyc A (Figure 2B). The adenine nucleotide translocator could not be involved in the activity of *Hemidesmus*. To demonstrate the opposite, the next set of experiments involved the use of ADP plus oligomycin, which acts on the adenine nucleotide translocator to inhibit pore opening. Oligomycin was added to avoid mitochondrial ADP conversion into ATP. ADP did not reduce the mitochondrial depolarization induced by *Hemidesmus* (Figure 2B). The mitochondrial depolarization of valinomycin, used as positive control, was significantly attenuated by all the inhibitors. The effect was particularly marked following treatment with ADP (data not shown).

### *Hemidesmus* indicus increases [Ca^2+^]_i_

Elevation of cytosolic Ca^2+^ is sufficient to induce mitochondrial permeability transition pore opening and brings to apoptosis in different cell systems [22]. We therefore studied the ability of *Hemidesmus* to modulate [Ca^2+^]_i_. Following 24 h-treatment with *Hemidesmus*, [Ca^2+^]_i_ was found to be about 5 times higher than that of the controls (Figure 2C). Short times of exposure (3 h) induced a smaller but still significant increase in [Ca^2+^]_i_ (Figure 2C). The recorded mean fluorescence intensity values clearly indicated two defined cell populations with different intracellular calcium levels (Figure 2D). In *Hemidesmus*-treated cells, the mean fluorescence values were 162.88 and 6.49 (*P*<0.01), respectively (Figure 2D).

**Figure 4:**
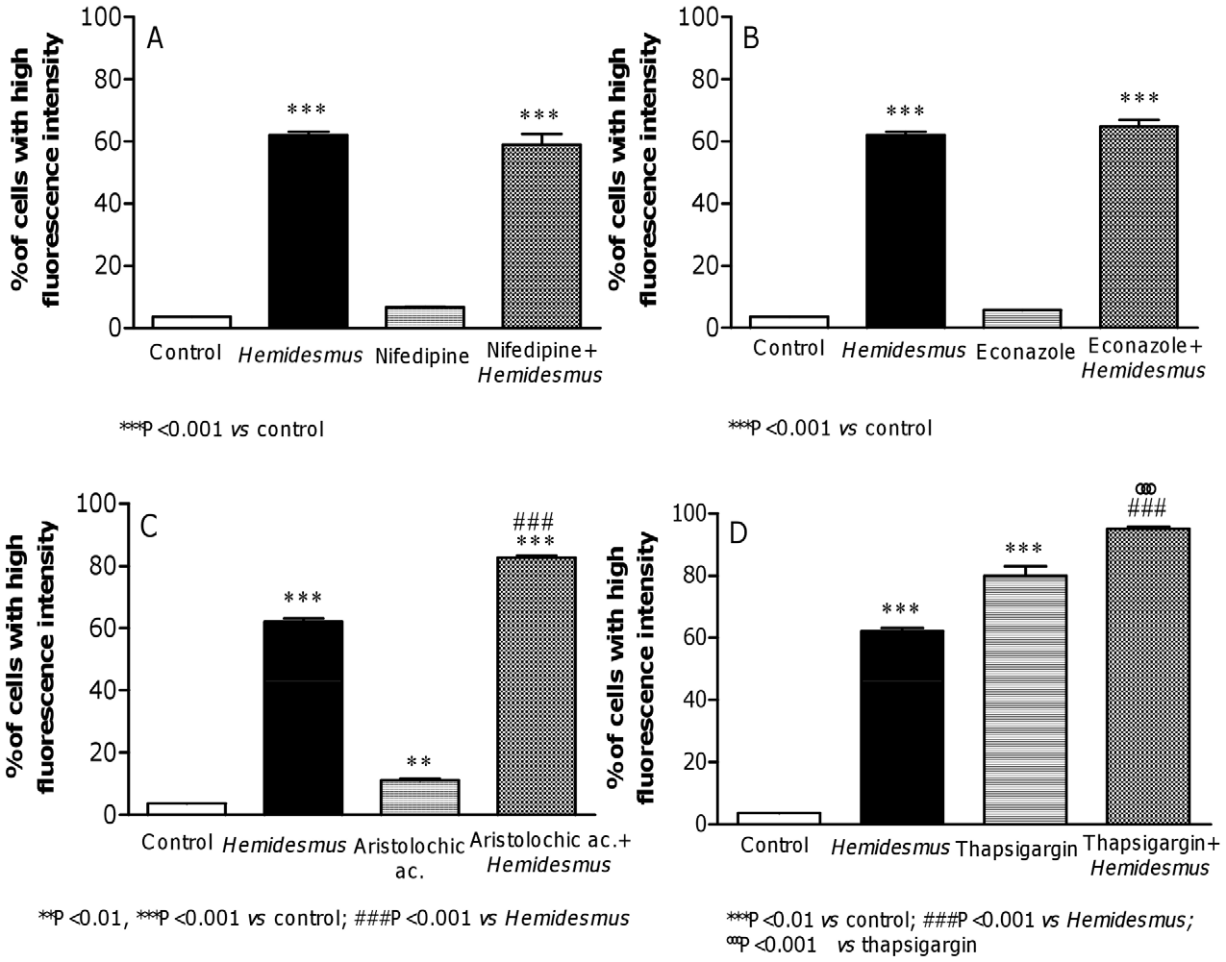
Effect of different inhibitors on *Hemidesmus*-induced [Ca^2+^]_i_ raise. Effects of nifedipine (A), econazole (B), aristolochic acid (C) and thapsigargin (D) on *Hemidesmus*-induced [Ca^2+^]_i_ raise. Data are means ± SEM of three independent experiments.

Experiments were performed to explore the pathway of *Hemidesmus*-induced [Ca^2+^]_i_ raise. The removal of extracellular Ca^2+^ did not abolish the [Ca^2+^]_i_ raise induced by *Hemidesmus* (data not shown). Some Ca^2+^ influx inhibitors, such as nifedipine and econazole, failed to affect *Hemidesmus*-induced [Ca^2+^]_i_ rise in Ca^2+^-containing medium (Figures 4A and B). In contrast, aristolochic acid and thapsigargin significantly increased *Hemidesmus*-induced [Ca^2+^]_i_ rise (Figures 4C and D).

### *Hemidesmus* indicus affects the expression of Bax and Bcl-2 protein levels

**Figure 5:**
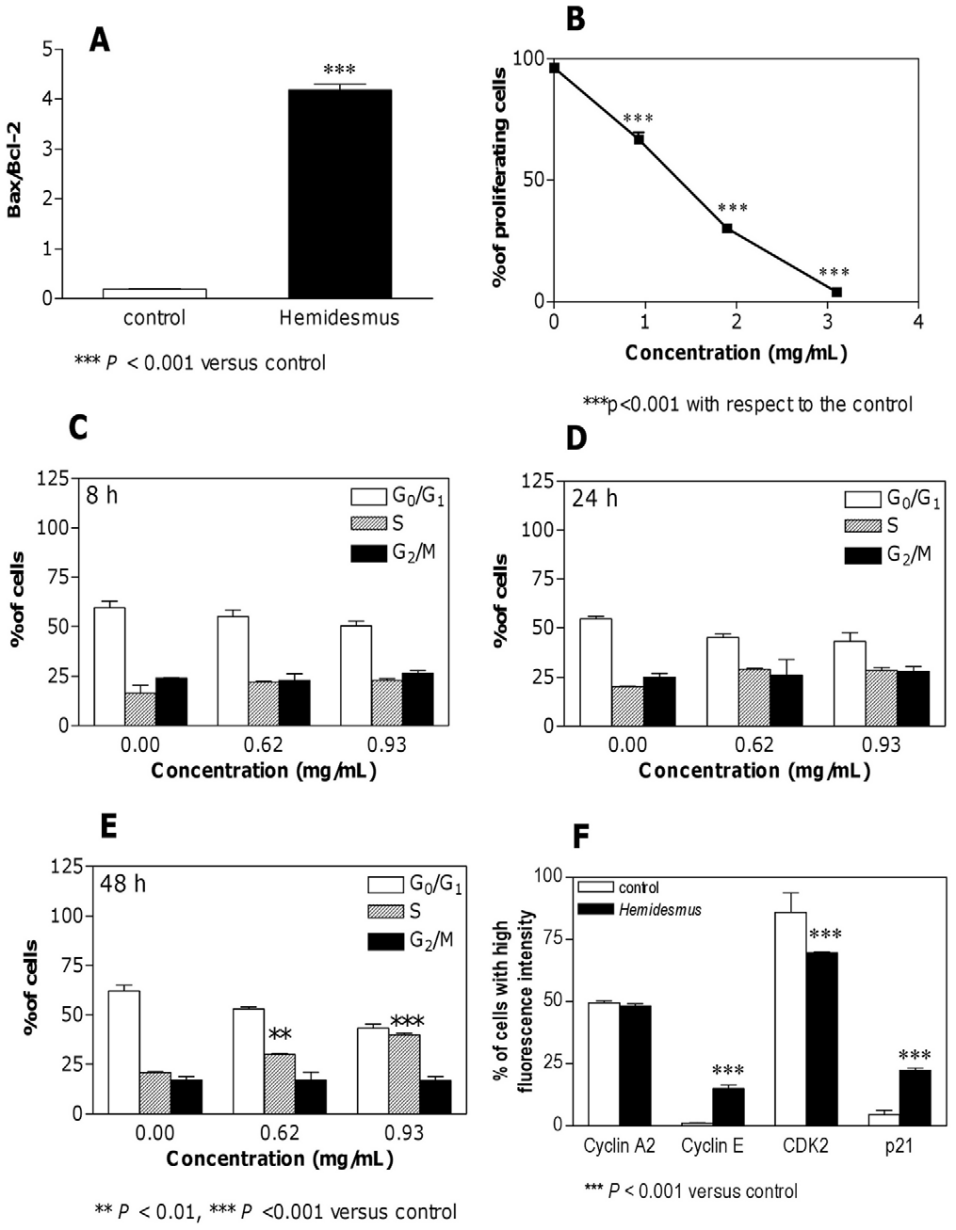
Effects of Hemidesmus on Bax-to-Bcl-2 ratio and cell proliferation. Bax-to-Bcl-2 ratio (A), cell proliferation (B), cell-cycle distribution (C–E), and cyclin A2, cyclin E, CDK2, and p21 protein levels (F) following 24 h culture in the absence or presence of *Hemidesmus*. Data are means ± SEM of four independent experiments.

To quantify Bax and Bcl-2 expression, the mean fluorescence intensity values were used. Both Bcl-2 and Bax expression were found to be significantly increased in treated cells relative to untreated cells (data not shown). Bax-to-Bcl-2 ratio was determined by using the mean fluorescence intensity value of *Hemidesmus*-treated cells that was normalized to the mean fluorescence intensity of untreated (control) samples within each group (Figure 5A). The Bax-to-Bcl-2 ratio was significantly higher than the control group (*P*<0.001).

### *Hemidesmus* indicus perturbs Jurkat cell proliferation

*Hemidesmus* significantly suppressed the progression of cells into the cell cycle (Figure 5B). At 0.93 mg/mL, *Hemidesmus* suppressed Jurkat proliferation by 30% and at 1.90 mg/mL by 66%. At 3.10 mg/mL, cell-cycle progression was almost completely suppressed. By looking at the fraction of live and dead proliferated and unproliferated cells, we observed that more than 90% of the cells were proliferated and dead (propidium iodide-positive) at the highest concentration of *Hemidesmus* tested (data not shown). These results indicate that the cytotoxicity of *Hemidesmus* against Jurkat cells was attributable to both the cytostatic effect causing the prevention of cell proliferation and the apoptotic and/or necrotic effect causing the loss of cell viability. Furthermore, these results confirm that low doses of *Hemidesmus* (0.93 mg/mL) could induce apoptotic cell death, but not necrosis. Since cell death represents the prevalent effect at the highest doses of *Hemidesmus* tested, the following analyses were limited to concentrations of up to 0.93 mg/mL.

### *Hemidesmus* indicus alters cell-cycle residence

At all concentrations, addition of *Hemidesmus* caused a dose-related accumulation of cells in the S phase (Figure 5C-E). The immediate effects (8 h) appeared primarily as an increase in the proportion of cells in the S phase of the cell cycle (from about 16% to 22%) accompanied by a slight compensatory decrease in G_1_ phase cells. Longer exposure (24 h) led to a further decrease in the proportion of G_1_ cells, while the percentage of cells in S phase increased from 20% to 29%. Prolonged (48 h) exposure appeared as a decrease in G_1_ phase cells (from 62% to 43%), an unaffected fraction of G_2_/M phase cells and a marked increase in the proportion of S cells (from 20 to 40%).

### *Hemidesmus* indicus affects the expression of cell-cycle regulatory proteins

Since *Hemidesmus* was found to selectively alter the distribution of Jurkat cells in the cell cycle, we evaluated its effects on the expression of cell regulatory proteins including cyclins A2 and E, CDK2 and p21. As shown in Figure 5F, the proteins specific for CDK2, cyclin A2, cyclin E, and p21 were easily detectable in continuously growing Jurkat cells. Among the protein levels, those of cyclin A2 remained relatively constant, whereas those of both cyclin E and p21 significantly increased after treatment with *Hemidesmus indicus* for 24 h (Figure 5F). Moreover, treatment with *Hemidesmus* greatly decreased the expression of CDK2 (Figure 5F).

### *Hemidesmus* indicus increases the antitumor efficacy of 6-thioguanine, cytarabine and methotrexate

**Figure 6:**
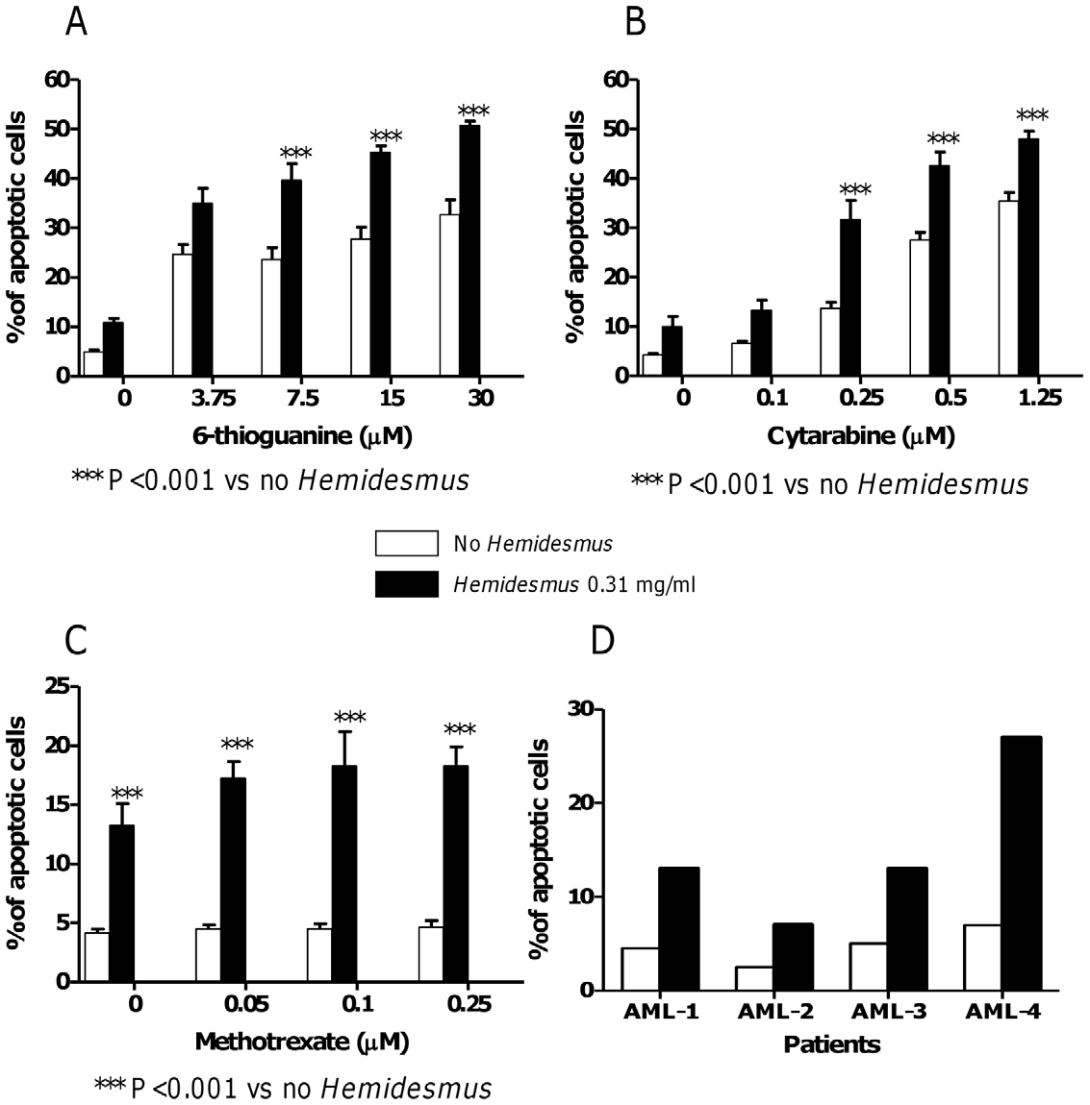
Induction of apoptosis by a combination of *Hemidesmus* and different anticancer drugs in Jurkat cells (A–C) and by *Hemidesmus* on primary cells (D). Fraction of apoptotic cells induced by 6-thioguanine (A), cytarabine (B) or methotrexate (C) following 24 h culture in the absence or presence of *Hemidesmus* (0.31 mg/mL) and fraction of apoptotic cells induced by *Hemidesmus* on mononuclear cells isolated from AML patients (D). Data on Jurkat cells are means ± SEM of six independent experiments.

To investigate whether *Hemidesmus* could increase the cytotoxicity of some anticancer drugs, cells were treated with a combination of 6-thioguanine, cytarabine or methotrexate plus *Hemidesmus*. We measured the proapoptotic effect of the combination using doses of *Hemidesmus* that induced submaximal toxicity (0.31 mg/mL). This can allow observing potential additive or synergistic effects. Combination of *Hemidesmus* with 6-thioguanine, cytarabine or methotrexate had a synergistic or additive proapoptotic effect compared with each drug present alone (data summarized in Figure 6A-C). For instance, when 6-thioguanine was used alone, a 33% of apoptotic cells (*versus* 5% in the untreated cultures, P<0.001) was observed at the highest concentration tested. When it was used together with 0.31 mg/mL of *Hemidesmus*, a 51% of apoptotic cells was induced (Figure 6A). Similarly, cytarabine alone at 1.25 µM induced a 35% of apoptotic cells (*versus* 4% in the untreated culture, P<0.001), but co-presence of *Hemidesmus* produced a 48% of apoptosis (Figure 6B). The CI was found to be 0.6 for 6-thioguanine and 0.38 for cytarabine.

The effect of *Hemidesmus* was also observed on cells treated with methotrexate. Treatment with methotrexate alone slightly affected cell viability. The % of apoptotic cells observed following treatment with methotrexate was similar to that of untreated cultures (Figure 6C). Because of the relative insensitivity of Jurkat cells to methotrexate, we could not calculate a CI. In this case, the interaction between *Hemidesmus* and methotrexate was classified using the fractional inhibition method as follows: when expressed as the fractional inhibition of cell viability, additive inhibition produced by both inhibitors (*i*) occurs when *i*_1,2_ =  *i*_1_ + *i*_2_; synergism when *i*_1,2_ > *i*_1_ + *i*_2_; antagonism when *i*_1,2_ < *i*_1_ + *i*_2_ [23]. Using this method, the interaction between *Hemidesmus* and methotrexate was additive.

### *Hemidesmus* indicus induces cytotoxic effects on mononuclear cells isolated from AML patients

We first estimated the effect of *Hemidesmus* on the viability of AML cells. *Hemidesmus* produced a cytotoxic effect in all samples tested. Viability of cells treated with *Hemidesmus* during 24 h decreased from 97.5% ±1.2 in the control to 55.0% ±3.1 in the cells treated with *Hemidesmus* (P<0.001). To assess whether cell death induced by *Hemidesmus* was due to apoptosis, we measured the exposure of membrane phosphatidylserine by flow cytometry. As shown in Figure 6D, *Hemidesmus* at 0.93 mg/mL induced a 3-fold increase in apoptotic cell fraction in patients AML-1, AML-2 and AML-3, and a 4-fold increase in AML-4 patient. Interestingly, the response to *Hemidesmus* was more pronounced on blasts obtained from a recidivant patient (AML4).

## Discussion

In this study, we show that *Hemidesmus indicus*, a traditionally-used medicinal plant, exerts potent antileukemic effects through the modulation of different critical targets. *Hemidesmus* was subjected to an HPLC analysis to quantify its main phytomarkers, namely 2-hydroxy-4-methoxybenzaldehyde, 3-hydroxy-4-methoxybenzaldehyde and 2-hydroxy-4-methoxybenzoic acid. We then examined its effect on human T-leukemia cell proliferation by focusing on cell-cycle regulation. According to our flow cytometry data, treatment of Jurkat cells with *Hemidesmus* resulted in a potent inhibition of cell growth, due to the block of cells in the S phase.

Many stimuli can induce cell arrest at different phases. Agents that cause damage to DNA or spindle apparatus will cause either apoptosis or cell-cycle arrest, which usually occurs at the G_1_/S or G_2_/M boundaries [24]. Moreover, certain taxanes and vinca alkaloids that cause G_2_/M arrest by damaging microtubules have proven clinically successful for cancer treatment [25]. Consequently, cell-cycle arrest at the G_1_/S and G_2_/M transitions has been intensively investigated in mammalian cells. In contrast, relatively little is known about mechanisms that control progress within the S phase. S-phase arrest has been observed in mammalian cells with prolonged arrest at the G_1_/S boundary [26]; Rb(+/+) mouse embryo fibroblasts treated with cisplatin, etoposide or mitomycin [27]; human melanocytes treated with thymidine dinucleotides [28]; and human osteosarcoma cells transduced with the p21 gene [29].

Because our study demonstrated that *Hemidesmus* treatment of Jurkat cells resulted in an S-phase delay, we examined the changes of some regulators associated with cell cycle in order to further elucidate the mechanism of action of *Hemidesmus*. The activation of preassembled replication complexes and initiation of DNA synthesis is mediated by cyclin A2/CDK2 complex and cyclin E/CDK2 complex. Cyclin A2 promotes both G_1_/S and G_2_/M transitions [30]. Cyclin A2/CDK2 activity is first evident in late G_1_, persists through S phase and peaks at G_2_ phase until prometaphase [31]. Cyclin E/CDK2 activity increases in late G_1_ and peaks in early S phase [32]. p21 is a CDK inhibitor that directly inhibits the activity of CDK2 activity [33].

*Hemidesmus* treatment resulted in a significant down-modulation of CDK2, whereas the expression of cyclin E was increased. Although *Hemidesmus* caused an increase in cyclin E levels, the global cellular response was a block of the cell cycle. In this situation, an important role can be played by p21, whose overexpression results in S-phase arrest [34]. *Hemidesmus* did not modulate the expression of cyclin A2. Since cyclin A2 is mainly involved in S progression and its expression peaks at G_2_ phase [30], this differential regulation suggests that the effect of *Hemidesmus* resides in early S phase.

*Hemidesmus*-treated cultures revealed a dose-dependent increase in the percentage of apoptotic cells. Apoptosis is primarily mediated through two pathways: the death receptor pathway and the mitochondrial pathway. In the death receptor pathway, a death receptor ligand, such as Fas ligand, binds to its receptor, such as Fas, triggering aggregation of the death receptor, recruitment of an adaptor molecule, such as FADD, as well as pro-caspase-8 or -10 forming a complex named the death inducing signaling complex. This results in the autocatalytic cleavage and activation of caspase-8 or caspase-10, leading to activation of caspase-3 or -7 and induction of apoptosis [35]. In the mitochondrial pathway, multidomain proapoptotic proteins, excessive mitochondrial calcium and reactive oxygen species induce the opening of the mitochondrial pore, with loss of mitochondrial integrity and transmembrane potential (Δ*Ψ*_m_) [36]. Cytochrome *c* is released from the mitochondria to form the apoptosome. The apoptosome then activates caspase-9, which in turn activates caspase-3, thereby inducing apoptosis. Many protein targets of active caspases are biologically important apoptotic indicators of morphological and biochemical changes associated with apoptosis [35]. One of the essential substrates cleaved by caspase-3 is PARP, an abundant DNA-binding enzyme that detects and signals DNA strand breaks [37].

In our system, *Hemidesmus* activated caspase-3 and induced PARP cleavage and cytochrome c release. The death receptor pathway was not induced by *Hemidesmus*, as indicated by the lack of activation of caspase-8. Thus, for the specific measurement of Δ*Ψ*_m_, Jurkat cells were loaded with the fluorochrome JC-1, a cationic probe that distributes passively between media, the cytosol and the mitochondria according to the Nernst’s equation, where the final distribution of the fluorochrome depends mainly on the transmembrane potential [38]. Compared to control cells, *Hemidesmus*-treated cells had drop in Δ*Ψ*_m_.

During the effector phase of mitochondria-dependent apoptosis, the inner transmembrane potential of the mitochondria collapses, indicating the opening of mitochondrial permeability transition pores. Mitochondrial permeability transition activation compromises the normal integrity of the mitochondrial inner membrane resulting into uncoupled oxidative phosphorylation, ATP decay, mitochondrial swelling and release of apoptogenic factors. The structure and composition of the transition pore includes inner membrane proteins, such as adenine nucleotide translocator, outer membrane proteins, such as the voltage-dependent anion channel, and cyclophilin D at contact sites between the mitochondrial outer and inner membranes [22]. The inner and outer membrane proteins operate in concert to create the conductance channels [35]. The adenine nucleotide translocator is an ADP-ATP antiporter that imports ADP to the matrix and exports ATP to the cytosol [39]. The adenine nucleotide translocator alternates between two distinct conformations in which adenine nucleotides are either bound to the cytosolic side (c-state) or to the matrix side (m-state) of the inner mitochondrial membrane [39]. CATR binds to adenine nucleotide translocator in the c-state. CATR binding occurs at a site similar to the ADP-binding site, thus preventing ADP/ATP transport. BA binds to adenine nucleotide translocator in the m-state. The two ligands are known to be adenine nucleotide translocator specific inhibitors [39]. The third putative component of the mitochondrial permeability transition is cyclophilin D, which binds to complexes of voltage-dependent anion channel and adenine nucleotide translocator in order to form the mitochondrial permeability transition complex. Cyc A was shown to block the binding of cyclophilin D [40].

To further elucidate the significance of mitochondria in *Hemidesmus*-induced Jurkat cell death, we investigated the effects of different inhibitors. *Hemidesmus* did not interact with adenine nucleotide translocator and did not disturb the effect of BA, CATR and Cyc A. The effect of *Hemidesmus* on mitochondrial depolarization was not even modulated by ADP. Because ADP is a potent ligand of the adenine nucleotide translocator [41], the results confirm that *Hemidesmus* does not stimulate the mitochondrial permeability transition through an interaction with the adenine nucleotide translocator but to an unrelated mechanism.

Numerous data have shown the pro-apoptotic effects of elevated concentrations of intracellular calcium [42] and, accordingly, many calcium ionophores are also apoptotic inductors in some cell types [38]. Since *Hemidesmus* possesses a strong proapoptotic effect in Jurkat cells, we were interested in studying its calcium mobilization activity. The major pathways of [Ca^2+^]_i_ increase are Ca^2+^ influx from extracellular space and Ca^2+^ release from internal Ca^2+^ stores. Numerous studies have demonstrated that both pathways appear to be involved in the [Ca^2+^]_i_ increase associated with apoptosis [43]. Our study demonstrated that *Hemidesmus* induced [Ca^2+^]_i_ rise and explored the underlying mechanisms.

Removal of extracellular Ca^2+^ did not abolish the [Ca^2+^]_i_ raise induced by *Hemidesmus*. Moreover, our results suggest that *Hemidesmus* did not cause Ca^2+^ influx via stimulating store-operated Ca^2+^ entry or voltage-gated Ca^2+^ channels because nifedipine (a blocker of L-type voltage-gated Ca^2+^ channels) [44] and econazole (an inhibitor of store-operated Ca^2+^ channels) [45] failed to inhibit the [Ca^2+^]_i_ raise. Aristolochic acid, a phospholipase A2 inhibitor, increased *Hemidesmus*-induced [Ca^2+^]_i_ raise. These findings indicate that phospholipase A2 could be not required for *Hemidesmus*-induced Ca^2+^ signal in our experimental model. However, aristolochic acid is able to induce a rapid rise in [Ca^2+^]_i_ through both release of endoplasmic reticulum stores and influx of extracellular Ca^2+^ [46]. To better understand the mechanism of *Hemidesmus*, we used thapsigargin, a compound that induces the release of intracellular endoplasmic reticulum Ca^2+^ stores and prevents refilling by inhibition of the endoplasmic reticulum Ca^2+^-ATPase [47]. Thapsigargin significantly increased *Hemidesmus*-induced [Ca^2+^]_i_ raise. On the whole, our results suggest that *Hemidesmus* may cause [Ca^2+^]_i_ raise through the mobilization of intracellular Ca^2+^ stores.

The integrity of the mitochondrial outer membrane is regulated by the Bcl-2 family. It has been shown that the Bcl-2 protein physically interacts with several of its homologous proteins. The most important interactions are considered to lie in Bcl-2/Bax dimerization. Thus, we studied the profile of Bcl-2 and Bax gene products in terms of protein expression. Our results showed that Bax gene expression was markedly induced, suggesting that Bax was upregulated and played an important role in the induction of apoptosis after *Hemidesmus* exposure. However, in contrast to the Bcl-2 inhibiting apoptotic cell death, the present study found that Bcl-2 expression was also increased after *Hemidesmus* exposure compared to control. The increase in anti-apoptotic Bcl-2 protein may serve as a compensatory protection of the leukemia cells upon *Hemidesmus* insult. Although the expressions of Bcl-2 and Bax, both of them, were increased, the ratio of Bax/Bcl-2 (pro- to anti-apoptotic proteins) was also increased after *Hemidesmus* treatment. The findings support the notion that the relative concentrations of pro-apoptotic and anti-apoptotic genes may act as a rheostat for the cell death program [48].

Altogether, our results suggest that the growth inhibition of Jurkat cells produced by *Hemidesmus* results from a combination of apoptosis and of cell-cycle derangements in which S accumulation is a key event. The *Hemidesmus*-induced S accumulation could have potentially important clinical implications. This factor made evaluation of the proapoptotic effects of *Hemidesmus* together with anticancer agents an important focus of our pre-clinical experiments. Cells which are synthesizing DNA usually display increased susceptibility to most anticancer drugs (*e.g.* antimetabolites or intercalating agents) [49]. The anticancer agents tested in this study included 6-thioguanine, cytarabine and methotrexate. Their inclusion was advantageous to determine whether *Hemidesmus* has the potential to be used as an adjunct to beneficially enhance the anticancer actions of certain clinically useful chemotherapeutic agents. Accordingly, the ability of *Hemidesmus* to increase the fraction of cells engaged in the S-phase of the cell division cycle was useful in potentiating the efficacy of 6-thioguanine, cytarabine and methotrexate.

In the last part of our study, we preliminarily explored the capacity of *Hemidesmus* to induce apoptosis in fresh AML cells in culture. Individualized tumor response testing *in vitro* of cells from patients with malignancies has been undertaken over the last 50 years [50]–[52]. These results have consistently shown that patients treated with drugs to which their cells were sensitive *in vitro* do significantly better clinically in terms of probability of response [53], [54], progression-free survival and overall survival [55]. We observed that *Hemidesmus* was a potent inducer of apoptosis in AML cells. Its proapoptotic activity was particularly marked on blasts obtained from a recidivant patient (AML4), characterized by a genomic alteration of the FLT3 gene, including FLT3/ITD. This observation is particularly interesting when we know that FLT3/ITDs are predictive of relapse and poor outcome in the chemotherapy setting [56].

Taken together, our results indicate that the traditional preparation of *Hemidesmus indicus* could be a promising botanical drug in the oncologic area. Since selective targeting and low toxicity for normal host tissues are fundamental requisites for anticancer drugs, it is interesting to note that our preliminary results indicated that the cytotoxic activity of *Hemidesmus* on non-transformed cells was significantly lower than that observed in cancer cells (unpublished work). This could allow the definition of a range of concentrations potentially active only on cancer cells.

*Hemidesmus* contains a mixture of known and possibly active compounds and this aspect poses many challenging issues. However, it is a relatively simple botanical product (single part of single plant). As a naturally occurring mixture from a single part of a single plant, it is not considered a combination product [6]. Of note, FDA determined that therapeutic consistency of the commercial batches of simple botanical preparations (single part of single plant) can be assured. Although the chemical constituents of a botanical drug are not always well defined and in many cases the active constituent is not identified nor is its biological activity characterized [7], variations in raw material quality can be minimized by restricting the cultivars and the composition of the preparation can be equivalent by robust chemistry, manufacturing and control measures, ‘fingerprinting’, conducting chromatographic analyses of marker compounds [6]. Our HPLC phytochemical analysis of *Hemidesmus indicus* demonstrated the presence of 2-hydroxy-4-methoxybenzoic acid, 2-hydroxy-4-methoxybenzaldehyde and 3-hydroxy-4-methoxybenzaldehyde, which can be used as fingerprint. Of note in this context, the phytochemical analysis performed on different batches of *Hemidesmus* demonstrated that the levels of the above reported phytomarkers were not statistically different among batches. The above reported phytomarkers lacked proapoptotic activities. Although relatively high doses of single bioactive agents may show potent anticarcinogenic effects, the antitumor properties of interactions among various ingredients that potentiate the activities of any single constituent may better explain the observed pharmacological effect of whole plant, as evidenced for other plants in many *in vitro* and *in vivo* studies [57]-[59].

On these bases, we conclude that the present plant can represent a valuable tool in the anticancer pharmacology, and should be considered for further investigations.
